# *Ultrabithorax* is a key regulator for the dimorphism of wings, a main cause for the outbreak of planthoppers in rice

**DOI:** 10.1093/nsr/nwaa061

**Published:** 2020-04-08

**Authors:** Fangzhou Liu, Xiang Li, Muhua Zhao, Mengjian Guo, Kehong Han, Xinxin Dong, Jing Zhao, Wanlun Cai, Qifa Zhang, Hongxia Hua

**Affiliations:** Hubei Insect Resources Utilization and Sustainable Pest Management Key Laboratory, College of Plant Science and Technology, Huazhong Agricultural University, Wuhan 430070, China; Hubei Insect Resources Utilization and Sustainable Pest Management Key Laboratory, College of Plant Science and Technology, Huazhong Agricultural University, Wuhan 430070, China; Hubei Insect Resources Utilization and Sustainable Pest Management Key Laboratory, College of Plant Science and Technology, Huazhong Agricultural University, Wuhan 430070, China; Hubei Insect Resources Utilization and Sustainable Pest Management Key Laboratory, College of Plant Science and Technology, Huazhong Agricultural University, Wuhan 430070, China; Hubei Insect Resources Utilization and Sustainable Pest Management Key Laboratory, College of Plant Science and Technology, Huazhong Agricultural University, Wuhan 430070, China; Hubei Insect Resources Utilization and Sustainable Pest Management Key Laboratory, College of Plant Science and Technology, Huazhong Agricultural University, Wuhan 430070, China; Hubei Insect Resources Utilization and Sustainable Pest Management Key Laboratory, College of Plant Science and Technology, Huazhong Agricultural University, Wuhan 430070, China; Hubei Insect Resources Utilization and Sustainable Pest Management Key Laboratory, College of Plant Science and Technology, Huazhong Agricultural University, Wuhan 430070, China; National Key Laboratory of Crop Genetic Improvement, Huazhong Agricultural University, Wuhan 430070, China; Shuangshui Shuanglü Institute, Huazhong Agricultural University, Wuhan 430070, China; Hubei Insect Resources Utilization and Sustainable Pest Management Key Laboratory, College of Plant Science and Technology, Huazhong Agricultural University, Wuhan 430070, China; Shuangshui Shuanglü Institute, Huazhong Agricultural University, Wuhan 430070, China

**Keywords:** *Hox*, *Ultrabithorax*, rice planthopper, wing polymorphism

## Abstract

Rice planthoppers, the most devastating rice pests, occur in two wing forms: the short-wing form for rapid population growth and long-wing form for long-distance migration, which together create the mechanism for outbreak. Here we show that *Ultrabithorax* (*Ubx)* is a key regulator for switching between the long- and short-wing forms of rice planthoppers. *Ubx* is expressed in both forewing and hindwing pads, which is different from the canonical model of *Ubx* expression. In brown planthoppers, expression of *Ubx* (*NlUbx*) is regulated by nutritional status of the rice host. High-quality young plants induce *NlUbx* expression leading to the short-wing form; low-quality ripe plants reduce *NlUbx* expression resulting in long-wing form. We also showed that *NlUbx* is regulated by the insulin receptors NlInR1 and NlInR2. The default expression of *NlInR1* inhibits *NlUbx* resulting in long-wings, while high-quality hosts induce *NlInR2* expression, which represses *NlInR1* thus promoting *NlUbx* expression to produce short-wings.

## INTRODUCTION

Rice planthoppers cause the most serious yield losses of rice crops globally among all the insects and diseases of rice [1,2]. Three planthopper species, brown planthopper (BPH) *Nilaparvata lugens*, white-backed planthopper *Sogatella furcifera*, and small brown planthopper *Laodelphax striatellus*, are the major groups of pests that frequently occur in most rice growing areas of the world [3]. In addition to direct feeding on the rice plants, these planthoppers are vectors for several rice viruses that also cause heavy crop losses [4–8].

**Figure 1.**
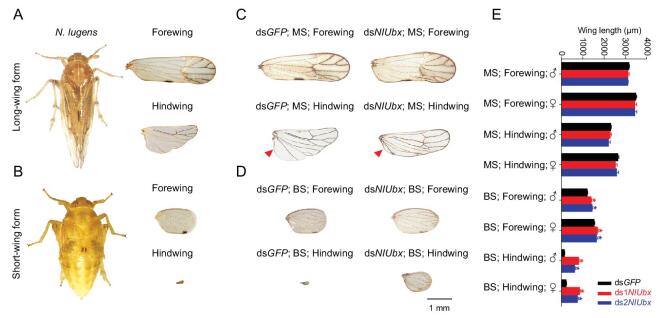
Wing morphology changes of *N. lugens* Macropterous Strain (MS) and Brochypterou Strain (BS) caused by ds*NlUbx*. (A,B) MS and BS of *N. lugens* from continuous selection in the laboratory. (C) Wings of *NlUbx*-RNAi MS adults compared with the ds*GFP* control. Bristles were ectopically formed on the hindwing, the proximal anal lobe of the hindwing was transformed to a clavus-like structure (indicated with red arrowheads), and the vein pattern of hindwing became more similar to that of the forewing. (D) Wings of *NlUbx*-RNAi BS insects compared with the ds*GFP* control. The number of bristles on the apical angle of the forewing was significantly increased compared to the control, and the wing veins and bristles were ectopically formed on the hindwing of *NlUbx*-RNAi BS adult. (E) Wing length alteration of *N. lugens* adults caused by *NlUbx*-RNAi, at least nine survival adults with phenotypic changes were measured. An ^*^ indicates a significant difference between the ds*NlUbx* and ds*GFP* treatments using a *t*-test (*P* < 0.05).

A very striking phenomenon, which comprises one of the most important causes for the outbreak of rice planthoppers, is the dimorphism of the wings. Rice planthoppers occur in two wing forms; the long-wing form has fully developed forewings and hindwings and the short-wing form bears severely reduced forewings and small bud-like hindwings (Fig. 1A and B). In areas where rice plants are at vegetative stage and the nutrition status is high, the short-wing form predominates so that the insect population can rapidly grow and reproduce [9,10]. When rice plants mature and the nutrition status becomes low, the long-wing form emerges to enable the insects to fly long distances to infest new rice fields [9,10]. While such dimorphism greatly increases both adaptability and fitness of the insects, which is of great interest in evolutionary biology, this strategy boosts the probability of large-scale disasters for rice production caused by the outbreak of the pests. Recent studies showed that two insulin receptors are key sensors of the nutritional status of the rice plants that ultimately determine the alternative wing morphs of BPH [11,12]. A natural question is thus whether there exists a key regulator that switches between the long- and short-wing forms in response to the nutritional signals.

There is enormous diversity in wing types among extant insect lineages. Based on the fossil records, the common ancestor of all winged insects appears to have two pairs of large membranous flight wings on its second and third thoracic segments (T2 and T3) [13]. Studies on the molecular basis of the differentiation of wing morphology in various insect lineages including dipteran, lepidopteran, hemipteran and coleopteran insects have suggested a canonical model, in which the Homeobox-containing gene (*Hox*) *Ultrabithorax* (*Ubx*) is the key regulator of wing development [14–17] and the key evolutionary factor that has driven lineage-specific wing differentiation [18–21]. Specifically, the current Hox-gene-based model of wing development posits that insect forewings are Hox-free structures and that *Ubx* functions as a ‘hindwing selector’ [22]. In *Drosophila melanogaster*, the forewing (on T2) is fully developed for flight, while the hindwing (on T3) is reduced to form a balancing structure called haltere. It has been revealed that *Ubx* in the developing hindwing negatively regulates many wing developing genes including *spalt*, *vestigial*, *serum response factor*, *knirps* and *achaete/scute* [15], and removal of *Ubx* alone in *Drosophila* is sufficient to transform the haltere structure to a membranous wing similar to the forewing [14]. Therefore, the key factor that reduces the hindwing to haltere is *Ubx* [14–17]. We thus hypothesize that the long/short wing transformation in the rice planthoppers may be regulated by *Ubx*.

In the present study, we demonstrated that *Ubx* is expressed in both forewing (T2) and hindwing (T3) pads in rice planthoppers, and functions as a master switch between short and long wings in response to host nutritional status.

## RESULTS

### *Ubx* regulates dimorphism in both forewings and hindwings in rice planthoppers

We conducted successive selection for over 40 generations, and obtained two strains of *N. lugens* that stably manifested the long-wing (∼85%) (referred to as Macropterous Strain, MS) and short-wing (∼100%) (Brachypterous Strain, BS) morphs, respectively (Fig. 1A and B). We prepared two RNA interference (RNAi) constructs (ds1*NlUbx* and ds2*NlUbx*) that target the coding region and 3’UTR of the *NlUbx* transcript sequence respectively (Supplementary Fig. 1A), and injected them into 3^rd^-instar nymphs of the MS and BS insects at the dosage of 200 ng/nymph. Very high mortality was observed in both ds1*NlUbx*- and ds2*NlUbx*-treated nymphs (80.8% and 95.7% mortality respectively) at seven days after microinjection (Supplementary Fig. 2A and B). We observed significant increases in the length of both forewing and hindwing in the survived *Ubx*-knockdown adults from BS (forewing increase by 7%–19%, hindwing increase by 240%–455%), and the increase was even more striking in the hindwing if measured by wing size (Supplementary Fig. 3). Such increase did not occur in MS.

**Figure 2.**
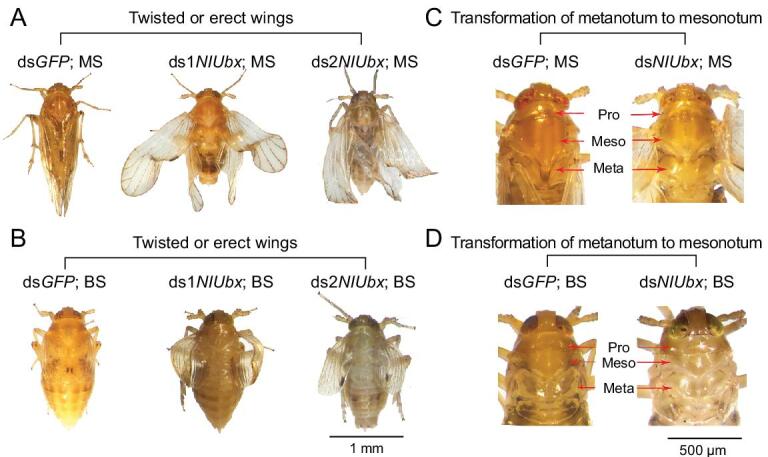
Morphological changes of *N. lugens* Macropterous Strain (MS) and Brochypterou Strain (BS) caused by ds*NlUbx*. (A) Twisted or erect wings of the *NlUbx*-RNAi MS adults. (B) Twisted or erect wings of the *NlUbx*-RNAi BS adults. (C) Alteration of the shape of metanotum to that of the mesonotum in the *NlUbx*-RNAi MS adults. (D) Alteration of the shape of metanotum to that of the mesonotum in the *NlUbx*-RNAi BS adults. Pro, Pronotum; Meso, Mesonotum; Meta, Metanotum.

Although there was no significant change in wing length in MS after *Ubx*-RNAi treatment, obvious morphological changes were observed in hindwing. This included the ectopic formation of bristles on the veins, reduction of the proximal anal lobe into a clavus-like structure (indicated by red arrowheads in Fig. 1C and Supplementary Fig. 4A and B), and the vein pattern which became more similar to the forewing (Fig. 1C). These changes made the hindwing morphologically similar to the forewing. The ds*NlUbx* treatment also caused other phenotypic changes in the BS besides wing length, including significant increase in the number of bristles on the apical angle of the forewing compared to the control (Fig. 1D and Supplementary Fig. 4C), and ectopic formation of wing veins and bristles on the hindwing (Fig. 1D and Supplementary Fig. 4D). These results suggested that the *Ubx*-RNAi treatment made the BS forewing more similar to the MS forewing, and the BS hindwing similar to the BS forewing. Moreover, there were other common morphological changes caused by *Ubx*-RNAi both in BS and MS, such as twisted or erect forewings and hindwings, as well as altering the shape of metanotum to that of the mesonotum (Fig. 2).

We also employed the RNAi approach to knockdown *Ubx* in the other two rice planthoppers using insects collected from the natural fields. *Ubx*-knockdown in *S. furcifera* and *L. striatellus* resulted in phenotypic changes similar to those observed in the ds*NlUbx* insects (Supplementary Figs 2, 5 and 6).

These results indicated that *Ubx* negatively regulates the length of the wings, which causes dimorphism in both forewings and hindwings in these rice planthoppers. The fact that reducing the expression of *Ubx* has pleiotropic effects on morphology seems to suggest that *Ubx* also has a role in the precise patterning of both forewings and hindwings in *N. lugens*.

### *Ubx* is expressed in both T2 and T3 in rice planthoppers

**Figure 3.**
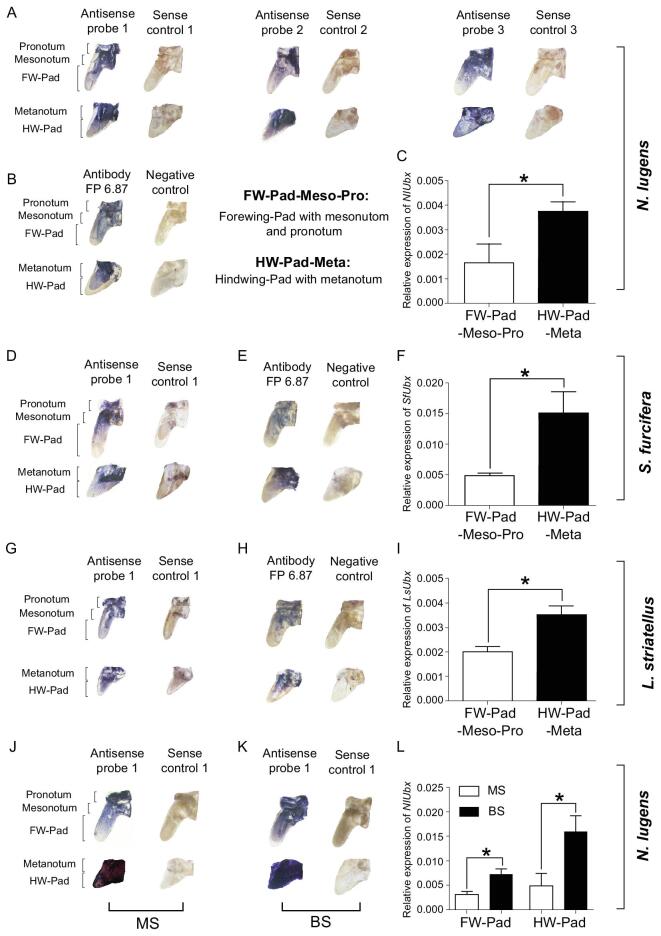
Expression analysis of *Ubx* in rice planthoppers. (A–C) *In situ* hybridization (A), immunohistochemistry staining (B), and qPCR (C) were used to detect *Ubx* expression levels in the thorax terga and wing pads of 5^th^-instar nymphs from a wild population of *N. lugens*. (D–F) *In situ* hybridization (D), immunohistochemistry staining (E), and qPCR (F) detection of *Ubx* expression levels in the thorax terga and wing pads of 5^th^-instar nymphs from wild population of *S. furcifera*. (G–I) *In situ* hybridization (G), immunohistochemistry staining (H), and qPCR (I) detection of *Ubx* expression levels in the thorax terga and wing pads of 5^th^-instar nymphs from wild population of *L. striatellus*. (J–L) *In situ* hybridization (J, K) and qPCR (L) detection of *Ubx* expression levels in the thorax terga and wing pads of 5^th^-instar nymphs from MS (Macropterous Strains) and BS (Brochypterous Strain) of *N. lugens*. An ^*^ indicates a significant difference in relative expression level between the tissues using a *t*-test (*P* < 0.05). FW: Forewing; HW: Hindwing. For qPCR analysis, ∼ 300 forewing pads or 400 hindwing pads were mixed for one replication, three replications for one tissue. *Actin1* was used as the reference gene to calculate the relative expression level of *Ubx*.

*Ubx* is generally assumed to be expressed only in T3 segment but not in T2 in a wide range of insect species, implying that it regulates hindwing development but not forewing [14–22]. Our result that suppressing the *Ubx* expression increased the length and caused other morphological changes of the forewings indicated that *Ubx* may also be expressed in T2 of the rice planthoppers. To examine whether this is the case, we analszed the expression of *NlUbx* in the thorax terga and wing pads of 5^th^-instar nymphs of BPHs collected from the fields using *in situ* hybridization and immunohistochemistry staining. Using three ∼125 bp antisense probes targeting *NlUbx* (Supplementary Fig. 1A), *in situ* hybridization revealed that anti-sense probes detected obvious *NlUbx* expression in the pronotum, the mesonotum, the metanotum, the forewing pad and hindwing pad, while the sense-probe controls showed no obvious hybridization signal (Fig. 3A). Immunohistochemistry staining using an Ubx antibody FP6.87 (Developmental Studies Hybridoma Bank) revealed a similar pattern for the accumulation of NlUbx protein in the forewing pad and hindwing pad (Fig. 3B). We also used three separate primer pairs to perform qPCR of the *NlUbx* expression patterns, and the specificity of *NlUbx* amplification was confirmed via cloning and sequencing of the PCR products. qPCR analysis supported the result that *NlUbx* is expressed in the pronotum, mesonotum, metanotum, the forewing pad and hindwing pad (Fig. 3C).

We also examined the expression of the *Ubx* orthologs in filed collected *S. furcifera* and *L. striatellus* insects, using *in situ* hybridization, immunohistochemistry staining and qPCR. We detected *SfUbx* and *LsUbx* expression in the pronotum, mesonotum, metanotum, the forewing pad and hindwing pad (Fig. 3D–I), which is similar to the patterns of *Ubx* expression in *N. lugens*. These results confirmed that the T2 expression of *Ubx* is common in rice planthoppers, which is very different from *Drosophila* and many other insects where *Ubx* is not expressed in T2 [14–22].

We subsequently assayed the laboratory-reared MS and BS of BPHs for *Ubx* expression in wing pads, and found that *NlUbx* was expressed in the forewing pads and hindwing pads of 5^th^-instar nymphs from both the MS and BS (Fig. 3J and K). In general, *NlUbx* expression was significantly higher in hindwing pads than in forewing pads in both strains (Fig. 3L). The level of *NlUbx* expression was significantly higher in the BS than in the MS in both forewing and hindwing pads (Fig. 3L). Such expression patterns suggest that the degree of reduction in the wing length in BS relative to MS is highly related to the *Ubx* expression level, indicating that *Ubx* repression effect on wing length is dosage dependent.

### Ectopic expression of *Ubx* from other species changed wing morphology of *Drosophila* in a dosage dependent manner

**Figure 4.**
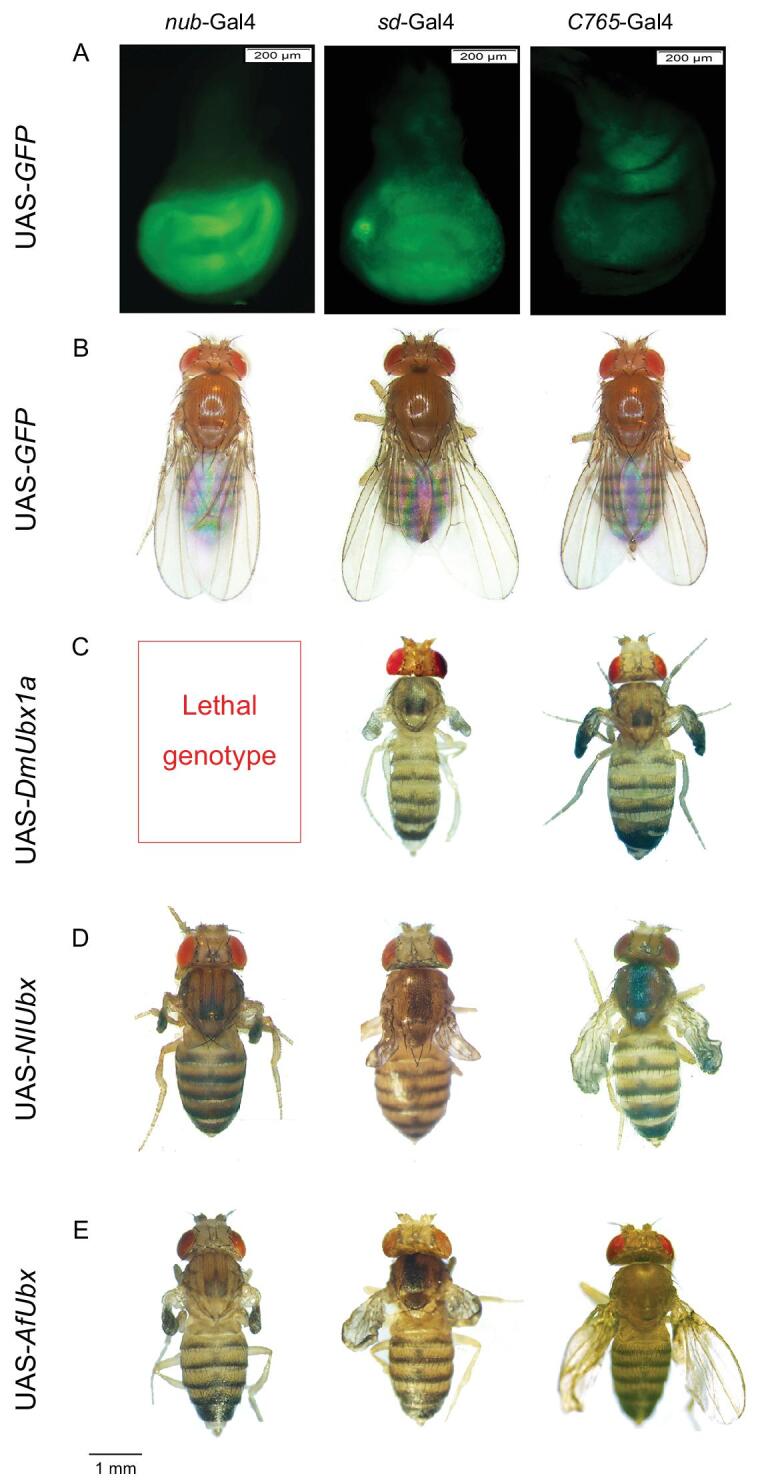
Effects of ecotopic expression of different *Ubx* genes on wing development of *Drosophila*. (A) Late 3^rd^-instar larval wing discs expressing *GFP* driven by *nub*-Gal4 (left), *sd*-Gal4 (middle), or *C765*-Gal4 (right). (B) Transgenic *GFP* flies grown at 18°C. (C–E) The wing phenotypes of adult flies expressing *DmUbx1a* (C), *NlUbx* (D), or *AfUbx* (E). *DmUbx1a*: *Drosophila melanogaster Ubx*; *NlUbx*: *Nilaparvata lugens Ubx*; *AfUbx*: *Artemia franciscana Ubx*.

To investigate whether ectopic expression of *Ubx*, in different dosages, may have effect on wing development in other insects, we ectopically expressed *Ubx* genes from different species of animals using the *Drosophila* model system. The *Drosophila* wing discs are anatomically similar to planthopper forewing pads and *Drosophila* haltere imaginal discs are similar to planthopper hindwing pads. Three different Gal4 enhancer trap fly lines [*nub*- (high dose) [23,24] *sd*- (intermediate dose) [25], and *C765*-Gal4 (weak dose) [25]] were employed to express three different *Ubx* orthologs controlled by Upstream Active Sequence (UAS): the dipteran *D. melanogaster Ubx* (*DmUbx1a*), the hemipteran *NlUbx*, and the crustacean *Artemia franciscana Ubx* (*AfUbx*), in *Drosophila* wing discs at three different dosages. The similarity of amino acid sequences of *NlUbx* and *DmUbx1a* is 46.8%, and that of *AfUbx* and *DmUbx1a* is 34.2%. We tested the system using a Green Fluorescent Protein (GFP) reporter in the developing wing disc of *Drosophila* larvae, which *per se* did not affect the wing development (Fig. 4A and B). It was shown that the fluorescence intensity of GFP varied with the Gal4 enhancer trap fly lines in accordance with the expected dosages, which quantitatively reflected the expression levels of ectopically expressed *Ubx* orthologs, although the insertion site of the UAS-*Ubx* construct in the genome might affect the expression level of *Ubx.*

The expression of *DmUbx1a* at three different dosages in *Drosophila* wing discs, which canonically does not express *Ubx*, resulted in three distinct phenotypes. The expression of *DmUbx1a* driven by the strong *nub-*promoter resulted in lethality (Fig. 4C, Supplementary Fig. 7A). The wing disc expressing *DmUbx1a* at intermediate dosage driven by the *sd*-promoter developed into a ‘wing-to-haltere like structure’, while the weak dosage of *DmUbx1a* driven by the *C765*-promoter caused a wing-to-haltere intermediate structure (Fig. 4C, Supplementary Fig. 7A). For the other two *Ubx* orthologs, the strong dosage of *NlUbx* and of *AfUbx* resulted in the development of wing-to-haltere like structures (Fig. 4D and E, Supplementary Fig. 7B and C). The intermediate dosage of *NlUbx* and *AfUbx* caused development of a small wing-like structure (Fig. 4D and E, Supplementary Fig. 7B and C). Finally, the wing disc with the weak dosage of *NlUbx* and *AfUbx* developed into a nearly intact wing, and the wing size of the flies expressing *AfUbx* was much larger than those expressing *NlUbx* (Fig. 4D and E, Supplementary Fig. 7B and C). These results indicate obvious dosage effects of the Ubx proteins on *Drosophila* wing size. The results also suggested that the degree of the repression may be related to the sequence similarities of the *Ubx* genes relative to the native *Drosophila Ubx* gene, such that the less similar *Ubx* gene has a less repressive effect on *Drosophila* wing development.

### The expression of *NlUbx* is regulated by host nutrition status

**Figure 5.**
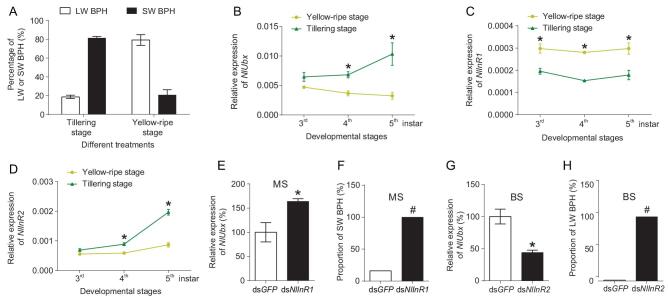
The responses of *NlUbx* to the host nutrition status and *NlInR1/R2*. (A) The proportion of long-wing or short-wing forms of adults reared on tillering stage (high-quality) or yellow-ripe stage (low-quality) rice plants. (B–D) Relative expression levels of *NlUbx* (B), *NlInR1* (C) and *NlInR2* (D) in the thorax terga and wing pads of 3^rd^-, 4^th^- and 5^th^-instar nymphs from wild population, which were reared with either high-quality or low-quality rice plants. (E) Expression level of *NlUbx* in the thorax terga and wing pads of 5^th^-instar nymphs from MS treated with ds*NlInR1*. (F) The proportion of SW adults emerged from nymphs of MS treated with ds*NlInR1*. (G) Expression level of *NlUbx* in the thorax terga and wing pads of 5^th^-instar nymphs from BS treated with ds*NlInR2*. (H) The proportion of long-wing adults emerged from nymphs of BS treated with ds*NlInR2*. *NlActin1* was used as the reference to calculate the relative expression levels of *NlUbx, NlInR1* and *NlInR2* in (B), (C) and (D). In (E) and (G) the expression levels of *NlUbx* in ds*NlInR1*- and ds*NlInR2*-treated insects were measured relative to that of the ds*GFP*-treated insects. An ^*^ indicates a significant difference between the high-quality and low-quality rice plants (B–D) or between the ds*NlInR1/2* treatment and the control (ds*GFP*) (E, G) using a *t*-test (*P* < 0.05). The **#** indicates a significant difference in the proportion of LW or SW adults between the ds*NlInR1/2* treatment and the control (ds*GFP*) (F, H) using Fisher exact test (*P* < 0.05). LW: Long-wing form; SW: Short-wing form. Around 50 thorax terga were mixed together for one replicate, three replicates for each treatment.

It is known that the nutritional quality of host rice plants upon which planthopper nymphs feed affects wing dimorphism (the proportion of long-wing versus short-wing adults) [9,10]. We thus hypothesize that the regulation of wing length by *Ubx* occurs in response to the nutritional status. To test this hypothesis, we reared field-collected *N. lugens* nymphs on either high-quality (tillering stage) or low-quality (yellow-ripe stage) rice plants. When the nymphs were reared on low-quality rice plants in the entire nymphal stage, 20.7% adults were of short-wing morph. In contrast, feeding on high quality rice plants in the whole nymphal stage led to the development of 81.3% adults to be of short-wing morph (Fig. 5A). These results are similar to previous reports [10]. To inquire whether the nutritional quality of the host affects the expression level of *Ubx*, we monitored the expression levels of *NlUbx* in thoracic pronotum and wing pad tissues of BPH nymphs feeding on high- or low-quality hosts using qPCR. It was found that before 4^th^-instar, *NlUbx* expression levels were not very different between the nymphs reared on low-quality plants and those on high-quality plants. Whereas, in 4^th^- and 5^th^-instar stages, *NlUbx* expression was significantly higher in the nymphs reared on high-quality host than that in the nymphs reared on low-quality plants, especially at 5^th^-instar stage (*P* = 0.03) (Fig. 5B). Thus, *Ubx* of the BPH nymphs was differentially expressed according to the nutritional status of the hosts, and the high-quality host could drastically induce the expression of *NlUbx* especially at the last nymphal stage (5^th^-instar).

### The expression of *NlUbx* is under the control of insulin signaling

A recent study demonstrated that two insulin receptors, NlInR1 and NlInR2, coordinately regulate wing morph in BPH [11]. The authors proposed that the long-wing form is the default developmental morph of planthoppers, whereby NlInR1 signaling inhibits the activity of the forkhead transcription factor NlFOXO, and the NlInR1 signaling eventually leads to long-wing adults, while the binding of NlInR2 to NlInR1 suppresses the NlInR1 signaling resulting in the short-wing form.

To investigate whether *NlInR1* and *NlInR2* are involved in the *Ubx*-mediated wing dimorphism regulation, we measured the expression levels of *NlInR1* and *NlInR2* in tissues containing thoracic pronotums and wing pads of the nymphs reared on the high- and low-quality hosts. The expression level of *NlInR1* was generally very low compared to *NlInR2*, although significant difference was detected between the two host treatments at the 3^rd^- to 5^th^-instar stages (Fig. 5C). *NlInR2* expression was not very different between the two host treatments at 3^rd^-instar, whereas in later stages, it was significantly enhanced by the high-quality host, especially at 5^th^-instar (*P* = 0.02) (Fig. 5D). The expression pattern of *NlInR2* in response to host nutrition quality corresponded well with *NlUbx.* Thus high quality nutrition at a later stage of nymph development increased *NlInR2* expression, which presumably suppressed the *NlInR1* signaling to produce short wings, consistent with the model of Xu *et al*. [11].

We decreased the endogenous expression of *NlInR1* and *NlInR2* by injecting ds*NlInR1* and ds*NlInR2*, and analysed the expression of *NlUbx* in the thoracic pronotum and wing pad tissues of 5^th^-instar nymphs. Knockdown of *NlInR1* in nymphs from the MS significantly increased the expression level of *NlUbx* (Fig. 5E), consequently all the resulting adults were of short-wing morph compared to 16.0% SW from the ds*GFP* treatment (Fig. 5F; Supplementary Fig. 8A and B). Knockdown of *NlInR2* in nymphs from the BS significantly decreased the expression of *NlUbx* (Fig. 5G), all the adults emerged from treated nymphs were of long-wing morph compared to 1.3% long wing from the ds*GFP* treatment (Fig. 5H). Interestingly, simultaneous interference of *NlInR1* and *NlUbx* in the nymphs from the MS strain resulted in adults with longer hindwings (Supplementary Fig. 8C, D and E). Other changes included more bristles on the apical angle of the forewing, ectopic bristles and wing veins on the hindwing (Supplementary Fig. 8C), compared to *NlInR1* single RNAi (Supplementary Fig. 8B). These results indicated that ds*NlUbx* partly abolished the ds*NlInR1* effects, also suggesting that *NlUbx* is downstream of *NlInR1* in regulating wing development.

FOXO is a downstream target of InR1 and InR2 [11]. We investigated the relationship between *NlUbx* and *NlFOXO*. Knockdown of *NlFOXO* in nymphs from BS and wild population had no effect on the expression of *NlUbx*, neither did knockdown of *NlUbx* influence the expression level of *NlFOXO*. This might be explained by the previous result that *NlFOXO* regulated wing development via phosphorylation not expression level [11].

## DISCUSSION

Rice does not grow in the winter in most rice producing areas. Although *N. lugens* only infests rice, it cannot overwinter in subtropics and temperate regions, which account for the majority of rice producing areas. Therefore seasonal long-distance migration to chase high nutrition food is an essential capacity for survivorship and reproduction of *N. lugens*. In the evolutionary process *N. lugens* acquired the ability to develop short or long wings in timely response to the nutrition status, either to quickly reproduce when nutrition quality of the rice plants is high, or to fly long distances to find new rice fields when the nutrition status is low. The results of the present study show that *Ubx* of rice planthoppers regulates the alteration of long and short wings by expressing in both T2 and T3 in response to the nutritional status of the host.

A previous study demonstrated that two insulin receptors play a key role in regulating wing morph type in BPH. Knockdown of *NlInR2* in BPH nymphs led to a strong bias towards long-winged morph adults while dysfunction of *NlInR1* resulted in a strong bias towards short-winged morph adults [11]. The present results extended the understanding by connecting NlInR1/R2 signaling with NlUbx, which may be summarized as the following. Nllp3 binding of NlInR1 activates the NlInR1 signaling leading to down-regulation of *NlUbx*, which forms the constitutive pathway for producing the long-wing form. It is likely that this pathway has evolved uniquely in the planthopper lineage, which facilitated the evolution of the wing polymorphism. Binding of NlInR1 by an excessive amount of NlInR2 induced by high nutrition of the rice host suppresses the NlInR1 signaling and consequently elevates the *NlUbx* level resulting in the short-wing morph. Thus while NlInR2 serves as the nutrition sensor in the NlInR1/R2 signaling pathway, the up and down of the *Ubx* function in response to the level of NlInR1 signaling provides a switch for the development of the long-wing and short-wing morphs of the rice planthoppers. This may be referred to as the *NlInR1/R2-NlUbx* pathway of wing morph regulation, although much of the detail has to be characterized in future studies.

Previous studies indicate that forewings are Hox-free structures and that *Ubx* functions as a ‘hindwing selector’ in a wide range of insects [14–22], including milkweed bugs [20], which also belong to Hemiptera, the same order as the rice planthoppers. We showed that *Ubx* is expressed in both T2 and T3 in rice planthoppers, which regulates the development of both forewing and hindwing and causes wing morph differentiation via dosage effects. It was previously reported that *Ubx* in honeybees is also expressed in both forewing and hindwing, with higher expression level in hindwing than in forewing [26], although it is yet unknown whether the honeybee *Ubx* has a functional role in forewing development. Nonetheless, these results suggest that the regulation of planthopper wing dimorphism by *Ubx* might represent a newly evolved (or apomorphic) state, thus a new mode of insect wing differentiation.

It should also be noted that in our study, the knockdown of *NlUbx* in the nymphs from BS did not fully transform the short-wing form into the long-wing form. This weaker phenotype may be explained by the fact that *NlUbx*-RNAi caused very high lethality, thus only weakly affected nymphs could survive to adulthood. There is also a possibility that other factor(s) may affect the wing dimorphism of planthoppers, which needs to be further investigated.

Our results showed that ecotopic expression of *Ubx* orthologs from *Drosophila*, *A. franciscana* and *N. lugens* in *Drosophila* resulted in wing-type to haltere-type transformation with the severity depending on the strengths of the promoters. Previous studies also showed that overexpression of *Ubx* of *Acanthokara kaputensis*, *Apis mellifera*, *Bombyx mori*, *Tribolium castenum* and *Drosophila* in the wing discs of *Drosophila* also transformed the wing toward a haltere [26–28]. These results suggested that *Ubx* genes from arthropods and their ancestors are functionally conserved in suppressing wing development and they might share the common target genes involved in wing development.

Based on the present results and data from the literature, *Ubx*-mediated insect wing development may be classified into three modes. The first mode is presumably found in the ancestral groups of insects that have two pairs of large membranous flight wings [13] where all wings are in *Ubx*-free state, because of either no expression of *Ubx* in T2 and T3 or no wing-repress activity. The second mode is represented by the case in *Drosophila*, in which the forewing (on T2) is fully developed for flight (*Ubx*-free), while the hindwing (on T3) is reduced to haltere due to *Ubx* expression [14–17]. The T3 expression of *Ubx* also specifies the development of diverse hindwing structures found in many lepidopterans [19,21], coleopterans [18] and hemipterans [20]. The third mode is exemplified in the present study, which showed that the change between long and short wings (wing dimorphism) of the rice planthoppers is due to the up- and down-regulation of *Ubx* in both T2 and T3 in response to nutritional conditions. We further speculate that changes of wing sizes by up- and down-regulation of *Ubx* in both T2 and T3 may be a general mechanism in insects with polyphenic wings, although much remains to be investigated in future studies.

The results also have implications for field management to reduce crop loss. Traditionally, deployment of resistance genes has been generally considered as the most economic strategy for combating the rice planthopper pests. These results suggest that interrupting the migration routes of the planthoppers by changing the rice cropping system on a large geographical scale to create missing links in the chain, such as the practice of adopting late rice to replace middle-season varieties in the rice–crawfish system which is now gaining popularity in south China, may provide new strategies for the control of rice planthoppers and thus deserve strong efforts of exploration.

## METHODS

Two strains of predominantly short-wing form (Brachypterous Strain, BS) and long-wing form *N. lugens* (Macropterous Strain, MS) were obtained by 40 successive generations of selection following the thoughts of Morooka and Tojo [29]. *Ubx* clonging, *in silico* analysis, synthesis of dsRNA and microinjection, qPCR, *in situ* hybridization, immunohistochemistry staining, ectopic expression of *Ubx* orthologs in *Drosophila,* rearing *N. lugens* nymphs on rice with different nutrition quality and data analysis were described in detail in the Supplementary Materials.

## Supplementary Material

nwaa061_Supplemental_File

## References

[1] Heong KL , Cheng J, Escalada MM. Rice Planthoppers. Hangzhou: Zhejiang University Press, 2015.

[2] Liu WC , Liu ZD, Huang C et al. Statistics and analysis of crop yield losses caused by main diseases and insect pests in recent 10 years. Plant Protect 2016; 42: 1–9.

[3] Heong KL , Hardy B. Planthoppers: New Threats to the Sustainability of Intensive Rice Production Systems in Asia. Los Banós: International Rice Research Institute, 2009.

[4] Rivera CT , Ou SH, Lida TT. Grassy stunt disease of rice and its transmission by *Nilaparvata lugens* Stål. Plant Dis Rep 1966; 50: 453–6.

[5] Ling KC , Tiongco ER, Aguiero VM. Transmission of rice ragged stunt disease. Int Rice Res Newsl 1977; 2: 11–2.

[6] Zhou GH , Wen JJ, Cai DJ et al. Southern rice black-streaked dwarf virus: a new proposed Fiji virus species in the family Reoviridae. Chin Sci Bull 2008; 53: 3677–85.

[7] Zhang YX , Wang Q, Jiang L et al. Fine mapping of qSTV11KAS, a major QTL for rice stripe disease resistance. Theor Appl Genet 2011; 122: 1591–604. 21384112 10.1007/s00122-011-1557-0 PMC3082044

[8] Wang BX , Jiang L, Chen LM et al. Screening of rice resources against rice black-streaked dwarf virus and mapping of resistant QTL. Acta Agron Sin 2010; 36: 1258–64.

[9] Kusakabe SI . Dispersal of the brown planthopper, *Nilaparvata lugens* Stål (Hemiptera: Delphacidae) in relation to its population growth. Appl Entomol Zool 1979; 14: 224–5.

[10] Hu DB , Luo BQ, Li J et al. Genome-wide analysis of *Nilaparvata lugens* nymphal responses to high-density and low-quality rice hosts. Insect Sci 2013; 20: 703–16. 23956011 10.1111/j.1744-7917.2012.01571.x

[11] Xu HJ , Xue J, Lu B et al. Two insulin receptors determine alternative wing morphs in planthoppers. Nature 2015; 519: 464–7. 25799997 10.1038/nature14286

[12] Lin X , Xu Y, Jiang J et al. Host quality induces phenotypic plasticity in a wing polyphenic insect. Proc Natl Acad Sci USA 2018; 115: 7563–8. 29967173 10.1073/pnas.1721473115 PMC6055199

[13] Grimaldi DA , Engel MS. Evolution of the Insects. Cambridge: Cambridge University Press, 2005.

[14] Lewis EB . A gene complex controlling segmentation in *Drosophila*. Nature 1978; 276: 565–70. 103000 10.1038/276565a0

[15] Weatherbee SD , Halder G, Kim J *et al.* *Ultrabithorax* regulates genes at several levels of the wing-patterning hierarchy to shape the development of the *Drosophila* haltere. Gene Dev 1998; 12, 1474–82. 9585507 10.1101/gad.12.10.1474 PMC316835

[16] Hersh BM , Nelson CE, Stoll SJ et al. The UBX-regulated network in the haltere imaginal disc of *D. melanogaster*. Dev Biol 2007; 302: 717–27. 17174297 10.1016/j.ydbio.2006.11.011 PMC1892158

[17] Struhl G . Genes controlling segmental specification in the *Drosophila* thorax. Proc Natl Acad Sci USA 1982; 79: 7380–4. 6961417 10.1073/pnas.79.23.7380 PMC347343

[18] Tomoyasu Y , Wheeler SR, Denell RE. *Ultrabithorax* is required for membranous wing identity in the beetle *Tribolium castaneum*. Nature 2005; 433, 643–7. 15703749 10.1038/nature03272

[19] Weatherbee SD , Nijhout HF, Grunert LW et al. Ultrabithorax function in butterfly wings and the evolution of insect wing patterns. Curr Biol 1999; 9: 109–15. 10021383 10.1016/s0960-9822(99)80064-5

[20] Medved V , Marden JH, Fescemyer HW et al. Origin and diversification of wings: insights from a neopteran insect. Proc Natl Acad Sci USA 2015; 112: 15946–51. 26668365 10.1073/pnas.1509517112 PMC4702999

[21] Tong X , Hrycaj S, Podlaha O et al. Overexpression of Ultrabithorax alters embryonic body plan and wing patterns in the butterfly *Bicyclus anynana*. Dev Biol 2014; 394, 357–66. 25169193 10.1016/j.ydbio.2014.08.020

[22] Tomoyasu Y . *Ultrabithorax* and the evolution of insect forewing/hindwing differentiation. Curr Opin Insect Sci 2017; 19: 8–15. 28521947 10.1016/j.cois.2016.10.007

[23] Calleja M , Moreno E, Pelaz S et al. Visualization of gene expression in living adult *Drosophila*. Science 1996; 274: 252–5. 8824191 10.1126/science.274.5285.252

[24] Zirin JD , Mann RS. Nubbin and Teashirt mark barriers to clonal growth along the proximal-distal axis of the *Drosophila* wing. Dev Biol 2007; 304: 745–58. 17313943 10.1016/j.ydbio.2007.01.025 PMC1945053

[25] Garaulet DL , Foronda D, Calleja M et al. *Polycomb*-dependent *Ultrabithorax* Hox gene silencing induced by high Ultrabithorax levels in *Drosophila*. Development 2008; 135: 3219–28. 18715947 10.1242/dev.025809

[26] Prasad N , Tarikere S, Khanale D et al. A comparative genomic analysis of targets of Hox protein Ultrabithorax amongst distant insect species. Sci Rep 2016; 6: 27885. 27296678 10.1038/srep27885 PMC4906271

[27] Grenier JK , Carroll SB. Functional evolution of the Ultrabithorax protein. Proc Natl Acad Sci USA 2000; 97: 704–9. 10639143 10.1073/pnas.97.2.704 PMC15394

[28] Pavlopoulos A , Akam M. Hox gene *Ultrabithorax* regulates distinct sets of target genes at successive stages of *Drosophila* haltere morphogenesis. Proc Natl Acad Sci USA 2011; 108: 2855–60. 21282633 10.1073/pnas.1015077108 PMC3041078

[29] Morooka S , Tojo S. Maintenance and selection of strains exhibiting specific wing form and body colour under high density conditions in the brown planthopper, *Nilaparvata lugens* (Homoptera: Delphacidae). Appl Entomol Zool 1992; 27, 445–54.

