# Supplementary material for: Ultrabithorax is a key regulator for the dimorphism of wings, a main cause for the outbreak of planthoppers in rice
Source: Natl Sci Rev. 2020 Apr 8;7(7):1181–9. doi: 10.1093/nsr/nwaa061 (PMC8288846; doi:10.1093/nsr/nwaa061)
Supplement: nwaa061_Supplemental_File [file nwaa061_supplemental_file.pdf]

Supplemental Materials for

Ultrabithorax is a key regulator for the dimorphism of wings, a main cause for the outbreak of planthoppers in rice

Fangzhou Liu[1], Xiang Li[1], Muhua Zhao[1], Mengjian Guo, Kehong Han, Xinxin Dong, Jing Zhao, Wanlun Cai, Qifa Zhang[2], Hongxia Hua[2]

[1]These authors contributed equally to this work.

[2]To whom correspondence should be addressed

Qifa Zhang

Email: qifazh@mail.hzau.edu.cn;

Hongxia Hua

E-mail: huahongxia@mail.hzau.edu.cn.

This PDF file includes:

Methods

References for Supplementary Materials

Supplementary Tables S1

Supplementary Fig. 1 to 8

Methods

Insects

Insects were raised with Taichuang Native 1 (a BPH-susceptible rice variety), in a growth chamber under conditions of 28±1°C, 14 h light/10 h dark and 70 ± 5% relative humidity. Wild specimens of *Nilaparvata lugens*, *Sogatella furcifera,* and *Laodelphax striatellus* were collected from paddy fields in Wuhan, Hubei Province, China, and reared in the lab for at least 5 generations before use ('wild population'). We also conducted experiments with predominantly short-wing forms (Brachypterous Strain, BS) or long-wing forms *N. lugens* (Macropterous Strain, MS) that were obtained by more than 40 successive generations of selection following the thought of Morooka and Tojo (1). Nearly 100% of the adults in the BS are short-wing phenotypes; ~85% of the adults in the MS are long-wing phenotypes.

Cloning of *Ubx*

Expressed sequence tags (EST) for *N. lugens Ubx* (*NlUbx*) were obtained from the transcriptome data for *N. lugens* wing pads (2). A rapid amplification of cDNA ends (RACE) method was used to isolate full-length cDNA of *NlUbx* using a Smarter RACE cDNA Amplification kit (Clonetech), according to the manufacturer's instructions. The full coding sequences (CDS) of *S. furcifera Ubx* (*SfUbx*) and *L. striatellus Ubx* (*LsUbx*) were amplified from total RNA based on EST sequences from their transcriptomes (sequenced in a project in our lab). Total RNA of the three planthopper species were extracted using Trizol reagent (Thermo Fisher Scientific, Cat.No.15596026). The primers used for EST cloning and RACE are shown in *SI Appendix,* Table S1.

In silico analysis

The sequence, start codon and stop codon of the protein were predicted using Expasy (http://web.expasy.org/). Functional domains of the protein were predicted using InterProScan (http://www.ebi.ac.uk/interpro/) (3).

Sample collection for analysis of *Ubx* expression

Thoracic pronotum and wing pad tissues of 5th-instar nymphs of *N. lugens* (including field-collected, MS and BS), *S. furcifera* and *L. striatellus* were collected using precision tweezers (WPI) under a stereoscope (Olympus szx10) for qPCR, *in situ* hybridization and immunohistochemistry staining. Total RNA of these tissues was isolated to examine the tissue-specific expression of *Ubx* using qPCR. These tissues were immediately put into Formalin-acetic acid-alcohol fixative (38% Formaldehyde: 5mL; Acetic acid: 5mL; 70% Ethanol: 90mL; Glycerine: 5mL; FAA) at 4°C, and pretreated for 12 h for *in situ* hybridization and immunohistochemistry staining.

Synthesis of dsRNA and microinjection

Synthesis of dsRNA and microinjection were conducted as described by Liu et al. (4). Two dsRNAs targeting the CDS or 3'-UTR of *NlUbx* were synthesized for gene knockdown of *NlUbx* (*SI Appendix*, Fig. 1*A*). dsRNAs targeting the CDS of *SfUbx* and of *LsUbx* were also synthesized for gene knockdown of *SfUbx* and *LsUbx* (*SI Appendix*, Fig. 1*B* and 1*C*). dsRNA sequences targeting *NlInR1/R2* were the same as Xu et al. (11). *GFP* (GenBank accession No.U76561) dsRNA (ds*GFP*) served as a negative control. The sequences of primers used to synthesize the dsRNAs are shown in *SI Appendix*, Table S1.

For *Ubx* RNAi, dsRNAs were injected into the 3rd-instar nymphs of *N. lugens, S. furcifera*, and *L. striatellus* at the dose of 200 ng/nymph. For *InR1*/*Ubx* double RNAi, 100 ng ds*NlInR1* and 200 ng ds*NlUbx* were injected into the 3rd-instar nymphs of *N. lugens* from MS. For each dsRNA injection, around 600 nymphs were used per replicate, with three replicates each. After injection, the survival rates of the treated planthoppers were recorded daily until adult emergence. Five nymphs treated with dsRNA were collected randomly on the 2nd and 4th day after injection for measuring the RNAi efficiency. Total RNA of the nymphs treated with dsRNA at 2 and 4 days after injection was extracted for qPCR analysis. When insects molted and adults emerged, phenotypes were observed under a stereomicroscope (Olympus szx16) and recorded. Wing length was measured from the base to tip under a stereomicroscope

(Olympus szx16) using cellSens Dimension 1.5 software. Twisted wings were unfolded by dipping in 5 μL ddH_2O on glass slides before measurement.

qPCR

qPCR analysis was conducted using the method of our previous studies (2, 4), using the primers listed in *SI Appendix*, Table S1. Independent reactions were performed in triplicate for each RNA sample, and three biological replicates were tested. The signal intensity of the target gene is presented as the average value. The housekeeping gene *Actin1* (Genbank accession number: *NlActin1*: EU179846.1, *SfActin1*: KP735520, *LsActin1*: KC683802.1) was used as the reference. The relative expression level of the gene was calculated according to the method of Livak and Schmittgen (5).

***In situ* hybridization**

The pronotum-mesonotum-forewing pads and metanotum-hindwing pads of the 5th-instar nymphs of planthoppers were collected from living tissue and immediately put into Formalin-acetic acid-alcohol fixative (38% Formaldehyde: 5mL; Acetic acid: 5mL; 70% Ethanol: 90mL; Glycerine: 5mL; FAA) at 4℃ for 12 h. Tert-Butanol was then used for decoloring of wingpads, followed by gradient alcohol dehydration. Tissue proteins were digested by Protease K, and tissues were then washed three times with 1 × Phosphate Buffered Saline (PBS). cDNA from *Ubx* was cloned into *pEASY*®-T3 Cloning Vector (TransGen Biotech, Cat.No. CT301-01) and used as template for PCR. Twenty bases of the T7 promoter were fused with *Ubx*-specific primers at the 5’ or 3’-end (T7-F and R or F and T7-R), so that the resulting PCR products contained the T7 polymerase promoter sequence at 5’ or 3’ ends. The PCR products were purified and used as the template for *in vitro* transcription; Digoxigenin (DIG)-labeled sense and antisense probes were synthesized using a DIG RNA Labeling Kit (SP6/T7) (Roche, Cat.No.1175025). *NlUbx* fragments were analyzed with 3 probes; *SfUbx* and *LsUbx* were each analyzed with a single probe. Whole-mount *in situ* hybridization of wing pads was conducted using a DIG-Nucleic Acid Detection Kit (Roche, Cat.No.11175041910) according to the manufacturer’s

instructions. Figures for *in situ* hybridization were taken under a stereomicroscope (Olympus szx16) using cellSens Dimension 1.5 software.

Immunohistochemistry staining

The pronotum-mesonotum-forewing pad and metanotum-hindwing pads were dissected in PBS and fixed with FAA fixative for 12 hours at 4°C. They were blocked in PBS, 5% BSA, 0.5% Triton for 30 minutes, incubated with the antibody (FP6.87, Developmental Studies Hybridoma Bank, http://dshb.biology.uiowa.edu/Ubx-ABD-A-FP6-87_2,) overnight at 4 ℃, washed 4 × in PBST (Phosphate Bussfer Saline Tween-20, pH=7.4) buffer and incubated with secondary antibody (Goat anti Mouse IgG, Thermo Fisher Scientific, Cat.No. 31430) coupled with Horseradish Peroxidase (HRP) for 1 hour at room temperature. They were then washed 4 times in PBST. Staining was achieved with 3,3’-diaminobenzidine (DAB) chromogen (between 5-10 min) and washing with PBST buffer.

Ectopic expression of *Ubx* orthologs in *Drosophila*

The three Gal4 enhancer trap lines and UAS-*GFP* fly line were provided by Dr. Shen Jie (China Agricultural University, Beijing). Full-length cDNA sequences were obtained by reverse transcription of the total RNA extracted from *D. melanogaster, N. lugens, and Artemia franciscana*. The primers for full-length cDNA cloning are shown in *SI Appendix*, Table S1. Final PCR products for the full *Ubx* CDS were gel purified, cloned into *pEASY*®-T3 Cloning Vector (TransGen Biotech, Cat.No. CT301-01), and subcloned into the pUAST vector (Provided by Core Facility of *Drosophila* Resource and Technology, Shanghai Institute of Biochemistry and Cell Biology, Chinese Academy of Sciences). UAS-*Ubx* constructs were transformed into *Drosophila* embryos using standard *P*-element-mediated germline transformation techniques (6). We crossed the *nub*-Gal4, *sd*-Gal4 and *C765*-Gal4 virgin females with UAS-*GFP* males and then put them in temperature-controlled incubators at 18 ℃. We used F1 late third-instar fly larvae for dissection to extract wing discs to observe the

fluorescence intensity of wing discs expressing *GFP*, which is driven by *nub*-, *sd*- and *C765* respectively. We also crossed the *nub*-Gal4, *sd*-Gal4 and *C765*-Gal4 virgin females with UAS-*Ubx* ortholog males and reared them at 18 ℃. The F1 adults were selected to observe wing phenotypes.

Rearing *N. lugens* nymphs on rice with different nutrition quality

The rearing experiment was conducted following Hu et al. (7) with minor modifications. The *N. lugens* wild population was used for this bioassay: 6 TN1 rice plants at the tillering stage (high-quality) or at the yellow-ripe stage (low-quality) were used to rear 500 neonates (< 12 h) until adult eclosion, the plants were refreshed when the nymphs reached 3rd instar stage. The rice plants were put into net cages (1 m long × 1 m wide × 0.9 m high), and the thoracic pronotum and wing pad tissues of 5th-instar nymphs (24-36 h after the molting climax) were sampled for gene expression analysis. Three replications were conducted for each rearing nutrition condition. The experiment was carried out under natural outdoor conditions in Wuhan in August 2018.

Data analysis

All data were analyzed using SPSS version 18, including survival response analysis after dsRNA microinjection, gene expression, the rate of LW or SW form and wing length. One-way analysis of variance (ANOVA) and LSD test, Student's *t*-tests (at $P < 0.05$) and Fisher exact test were used for comparison of differences between means.

Reference

1. Morooka S and Tojo S. Maintenance and selection of strains exhibiting specific wing form and body colour under high density conditions in the brown plant-hopper, *Nilaparvata lugens* (Homoptera: Delphacidae). *Appl Entomol Zool.* 1992; **27**, 445.
2. Li KY, Hu DB and Liu FZ *et al.* Wing patterning genes of *Nilaparvata lugens* identification by transcriptome analysis, and their differential expression profile in wing pads between brachypterous and macropterous morphs. *J Integr Agr.*

2015; **14**, 1796-807.

3. Zdobnov EM and Apweiler R. InterProScan–an integration platform for the signature-recognition methods in InterPro. *Bioinformatics.* 2001; **17**, 847-8.
4. Liu F, Li K and Li J *et al.* Apterous A modulates wing size, bristle formation and patterning in *Nilaparvata lugens*. *Sci Rep*. 2015; **5**: 10526.
5. Livak KG and Schmittgen TD. Analysis of relative gene expression data using real-time quantitative PCR and the $2^{-\Delta\Delta CT}$ method. *Methods.* 2001; **25**, 402-8.
6. Fischer JA, Giniger E and Maniatis T *et al.* GAL4 activates transcription in Drosophila. *Nature*. 1988; **332**: 853-6.
7. Hu DB, Luo BQ and Li J *et al.* Genome-wide analysis of *Nilaparvata lugens* nymphal responses to high-density and low-quality rice hosts. *Insect Sci*. 2013; **20**: 703-16.

Supplementary Tables

Supplementary Table 1 Primers used in this study

**Gene (Accession No.)**	**Name of primer**	**Sequence of primer (5'-3')**
NlUbx (KR869786)	primers for EST cloning	F: GTTGGTTATTGTTGGACACCCTC
		R: GCGTGATGAGACACAGAGATAATG
	primers for 5'-RACE	Out primer: GTGGTGGTGTTGACTGCTTGCGTAGGGT
		Inner primer: GTCTGGTGATGGTGACCTCCGTAGAAGC
	Primers for 3'-RACE	Out primer: CAGACGTACACGCGATACCAAACGCTG
		Inner primer: TGACGAGGAGACGGAGAATCGAGATGG
	Primers for ds1*NlUbx* synthesis	Sense: TAATACGACTCACTATAGGGTATGATGCTTCCGTGGCTG
		Antisense: TAATACGACTCACTATAGGGCAGGGGTAGAATGTGTGGTTG
	Primers for ds2*NlUbx* synthesis	Sense: TAATACGACTCACTATAGGGCCCAAAGACTCATAGCAGACAT
		Antisense: TAATACGACTCACTATAGGGGTAGATGAGGGGAAGGGAGAT
	Primers for qPCR	F1: GGATGTCACCCTACGCAAGCA
		R1: GCAGCCACGGAAGCATCATAC
		F2: GTGATTGAGTGAAGTTGGTT
		R2: AATGATGGTGGTGGTGTT
		F3: TAGCAGGAGCAAATGGCAT

		R3: CTAGTGGTCCGCCTGTTG
	Antisense probes for *in situ* hybridization (The sense probes are the reverse complementary sequence of antisense probes)	Antisense probe 1: GCGGCTGCGTAGCCGTTCTGATGGGCGCTCTCCGACTTATTACAATCTGGTTTGCTCGACGTGCTGTAGTTGATACTGCTCTGTTGCCCGGTATCATTGGAGCTAGAGTATAACTTGCAGGCTGCAG
		Antisense probe 2: TGATTGAAGTTAGAACGAGAGAGAGAGAGATAGCAGTGTGCCATACCTTTGACAGTCGTTGACTTTGGTGTTGACTGCGCCAACTGGTTGGCTGCATAAATCCAACATGGCGGCCAAAATATATTTATATAAAAAACAACACACACTCGCGCACTATTTGACACCGGATAGTATGTATATAGTATGATCATCACAATTGAACGGAAAAGTGGGTCTGTGGCATGTCTGCTATGAGTCTTTGGGTCCACCTCGCCGTGCGCCTGGTGGAGCAC
		Antisense probe 3: TGATTGAAGTTAGAACGAGAGAGAGAGAGATAGCAGTGTGCCATACCTTTGACAGTCGTTGACTTTGGTGTTGACTGCGCCAACTGGTTGG
	Primers for full-CDS cloning	F: CGGAATTCCGGTTGGTTATTGTTGGACACCCTC
		R: GCTCTAGAGCGCGTGATGAGACACAGAGATAATG
SfUbx (KY026484)	Primers for ds*SfUbx* synthesis	Sense: TAATACGACTCACTATAGGGAGACAGAGCAGTATCAACTACAGCACAT
		Antisense: TAATACGACTCACTATAGGGAGAGTGTGGTTGGAAGGTTGCTGA
	Primers for qPCR	F: TTTGAACAGTCGGGCTTCTA
		R: GCAGTGAATGATGGTGATGG
	Antisense probe for *in situ* hybridization (The sense probe is the reverse complementary sequence of antisense probe)	Antisense probe: ATAGCAGGAGCGAATGGCATGCGCCGGAGAGGCCGACAAACGTACACGCGATACCAAACCCTGGAGCTGGAGAAGGAGTTCCACACCAACCACTACCTGACGAGGAGACGACGCATCGAGATGGCGCACGCTCTCTGTCTCACCGAGCGACAGATCAAGATCTGGTTCCAGAACA

	Primers for full-CDS cloning	F: AACTGACGATAAGACGAGATGATGTG
		R: CATACCTTGGACATTCGTTGACTTT
LsUbx (KY026485)	Primers for ds*LsUbx* synthesis	Sense: TAATACGACTCACTATAGGGAGACAGAGCAGTATCAACTACAGCACAT
		Antisense: TAATACGACTCACTATAGGGAGAGTGTGGTTGGAAGGTTGCTGA
	Primers for qPCR	F: TTGGGAATGAGTCCGTATGC
		R: TTGCTCGATGTGCTGTAGTT
	Antisense probe for *in situ* hybridization (The sense probe is the reverse complementary sequence of antisense probe)	Antisense probe: TTCCCAACCCAAGTGGGAACCTATAAGCTGCAGCCGCAGCTGTCTGGTCGTGATGGTGTCCCGTTGCAGCTCCAGCTGTCTGATGATGGTGTCCGCCGTAAAAGCCCGACTGTTCAAAGTAAGAGTTCAT
	Primers for full-CDS cloning	F: AGAGACAGTGATTGTAGGTTGGTTATTG
		R: CTTGGACAGTGTTGACTTTGGTG
GFP (AY151052.1)	Primers for ds*GFP* synthesis	Sense: TAATACGACTCACTATAGGGGTGGAGAGGGCGAAGGTGATG
		Antisense: TAATACGACTCACTATAGGGATTTCCAAGGATGTTTCCATCTTC
NlActin1 (EU179846.1)	Primers for qPCR	F: CCAACCGTGAGAAGATGACC
		R: GATGTCACGCACGATTTCAC
SfActin1 (KP735520)	Primers for qPCR	F: AGAATGCAGAAGGAAATCAC
		R: ACGAACAGCAAATAAGTAGAAT
LsActin1 (KC683802.1)	Primers for qPCR	F: GACTCAGCCAGTGTTGAATCA
		R: CACAGCAGACAAACCAAGGA

NlInR1 (KF974333.1)	Primers for ds*NlInR1* synthesis	Sense: TAATACGACTCACTATAGGGAGACGCTCTGGTTGTGCTTGATA
		Antisense: TAATACGACTCACTATAGGGAGACGTTGTCTTTCTCCAACGGT
	Primers for qPCR	F: GAGTGCAACCCGGAGTATGT
		R: TCTTGACGGCACACTTCTTG
NlInR2 (KF974334.1)	Primers for ds*NlInR2* synthesis	Sense: TAATACGACTCACTATAGGGAGAAGCTGTGCAGGGAGAATGTT
		Antisense: TAATACGACTCACTATAGGGAGAAGTCCCTTGGTACCTTTCCG
	Primers for qPCR	F: CTCTTGCCGAACAGCCTTAC
		R: GGGTCGTTTAGTGGGTCTGA
AfUbx (AF435787.1)	Primers for full-CDS cloning	F: AGTGAAGTGATTTCAATCTTGTGCCCAC
		R: TCGGCACTTCCAACAAAGGAGCATT
DmUbx1a (FBpp0082797)	Primers for full-CDS cloning	F: AAAGGAGGCAAAGGAACAGCACA
		R: GTCATTTCGCCCAATCCCACATACA

Note: T7 sequences in the dsRNA synthesis primers were underlined.

Supplementary Figures

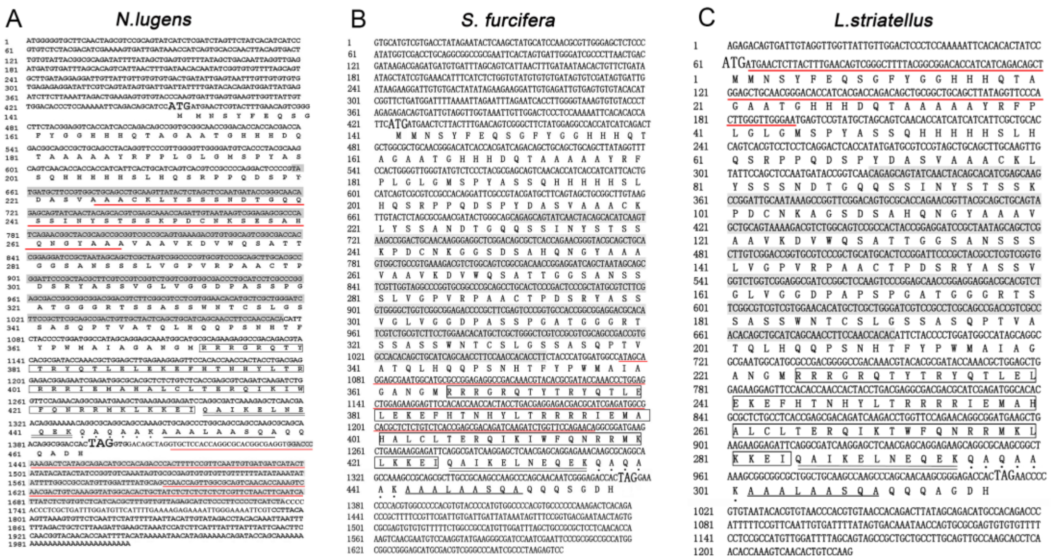

Supplementary Fig. 1 | Nucleotide and deduced amino acid sequences of *Ubx* genes from rice planthoppers. The start codon (ATG) and the stop codon (TAG) are in bold. The corresponding sequences of ds*Ubx* are shaded in grey. The corresponding sequences of anti-sense probes for *in situ* hybridization are indicated with red underlines. Homeobox domain is boxed. The Ubd-A peptide domain is labeled with double underline. QA motif is labeled with dot (…). Poly-Alanine repression domains are labeled with black underline.

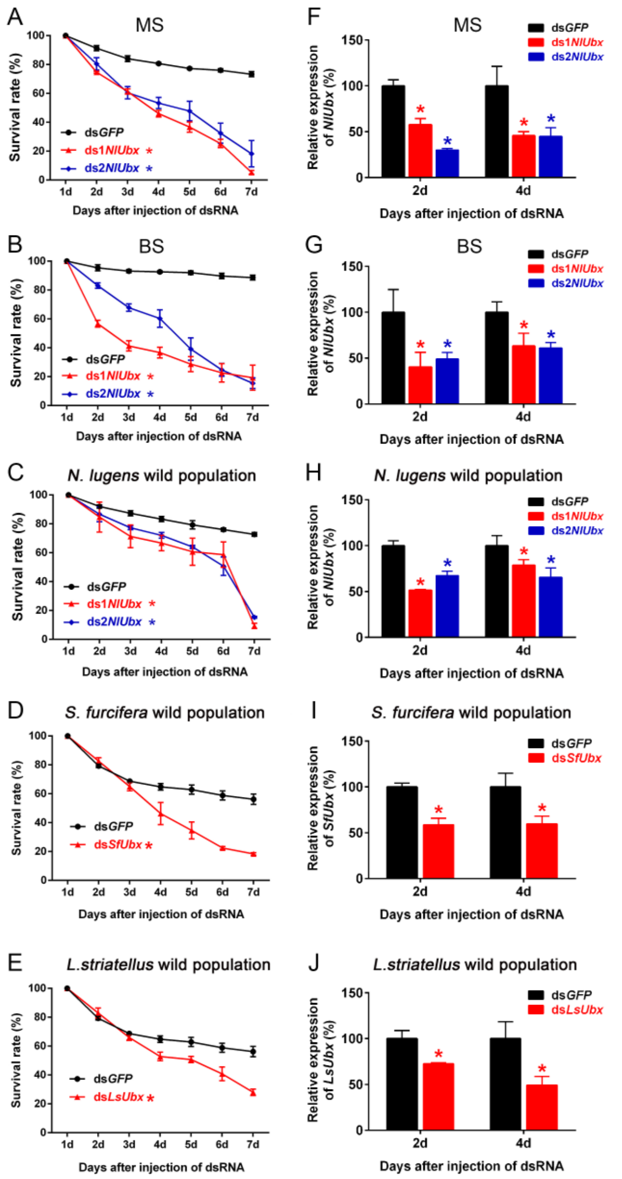

Supplementary Fig. 2 | Survival rates and the relative expression levels of *Ubx* in *N. lugens*, *S. furcifera* and *L. striatellus* after dsRNA injection. (A-C) Survival rates of the *N. lugens* nymphs from lab-reared Macropterous Strain (MS) (*A*), Brachypterous Strain (BS) (B) and the wild population (C) after ds1*NlUbx* and ds2*NlUbx* injections at 3rd instar. (D-E) Survival rates of *S. furcifera* and *L. striatellus* nymphs after ds*SfUbx* (D) and ds*LsUbx* (E) injection respectively at 3rd instar. (F-H) The relative expression levels of *NlUbx* in *N. lugens* of MS (F), BS (G) and wild population (H) after injection of ds1*NlUbx* and ds2*NlUbx* respectively at 3rd instar. (I-J) The relative expression levels of *SfUbx* in *S. furcifera* (*I*) and *LsUbx* in *L. striatellus* (J) after injection of ds*SfUbx* and ds*LsUbx* injection respectively at 3rd instar. ds*GFP*

injection was used as control. An * indicates significant difference ($P < 0.05$) between the ds*Ubx* treatment and ds*GFP* treatment on the same day using a *t*-test ($P < 0.05$). The mean ± SE was based on three biological replicates. For quantifying the expression of the *Ubx*, *Actin1* was used as the reference gene, and the expression level was measured relative to that of the ds*GFP* insects. For *Ubx* expression level analysis, 5 nymphs were mixed as one replicate, and 3 replicates for each treatment at each time point. For survival rate analysis, ~600 nymphs were injected with dsRNA for one replicate, 3 replicates for each treatment.

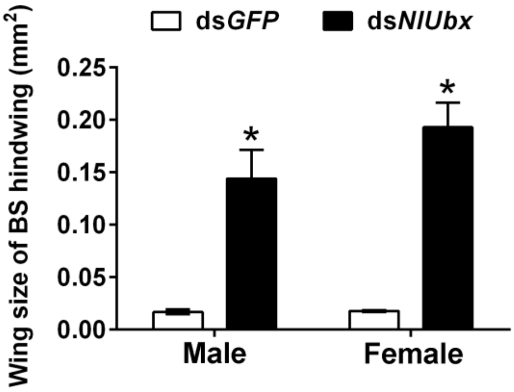

Supplementary Fig. 3 | The hindwing size of adults emerged from 3rd-instar nymphs of Brachypterous Strain treated with ds*GFP* and ds*NlUbx*. At least 9 survival adults with phenotypic changes were measured. An * indicates significant difference (P < 0.05) between the ds*NlUbx* treatment and ds*GFP* treatment using a *t*-test (P < 0.05).

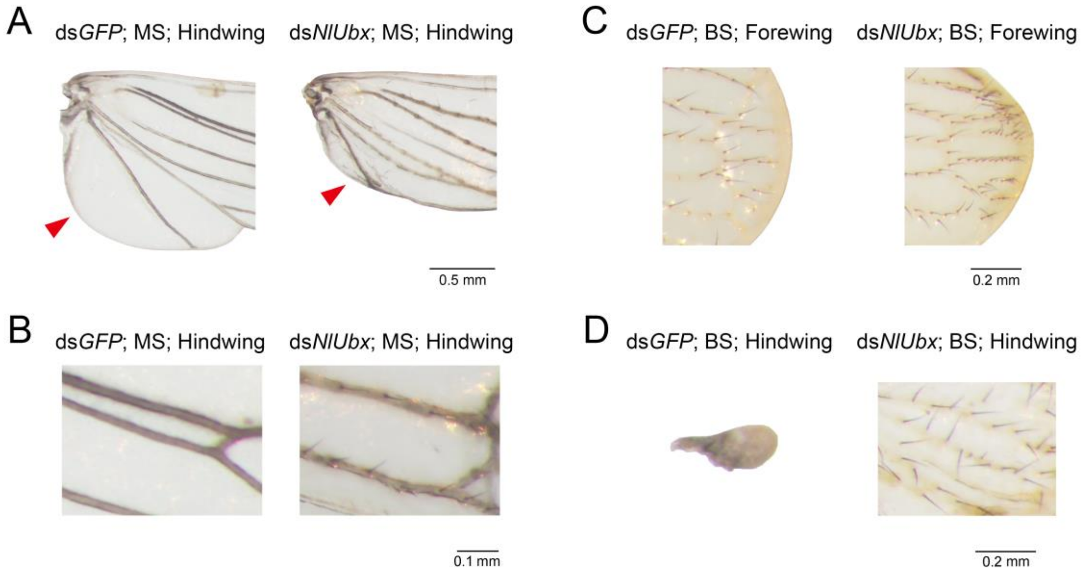

Supplementary Fig. 4 | Close-up pictures of ds*NlUbx* transformed wings. (A) transformation of proximal anal lobe into a clavus-like structure of the hindwing (indicated by red arrowheads) o*NlUbx*-RNAi MS adults. (B) the ectopic formation of bristles on the veins of the hindwing of *NlUbx*-RNAi MS adults. (C) increase in the number of bristles on the apical angle of the forewing of *NlUbx*-RNAi BS adults. (D) ectopic formation of bristles on the veins of the hindwing of *NlUbx*-RNAi BS adults. MS: Macropterous Strain; BS: Brachypterous Strain

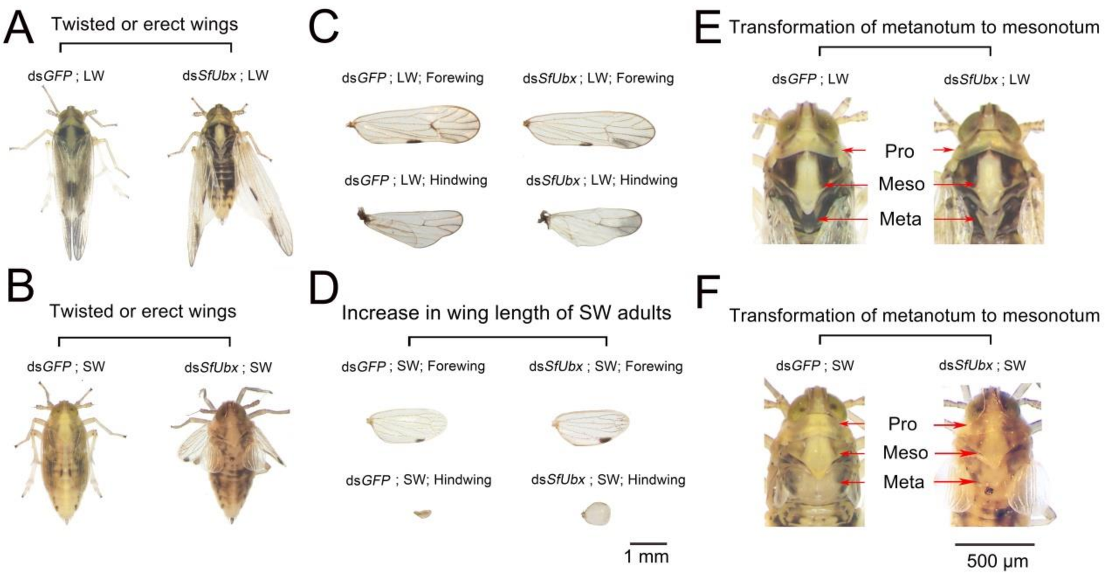

Supplementary Fig. 5 | Phenotypes of *S. furcifera* adults resulting from ds*SfUbx* injection at 3rd instar. (A) Twisted or erect wings in the long-wing form (LW). (B) Twisted or erect wings in the short-wing form (SW). (C) Forewing and hindwing of *S. furcifera* LW adults treated with ds*SfUbx*. (D) Forewing and hindwing of *S. furcifera* SW adults treated with ds*SfUbx*. (E) Transformation of metanotum to mesonontum in the LW. (F) Transformation of metanotum to mesonotum in the SW. Pro, Pronotum; Meso, Mesonotum; Meta, Metanotum.

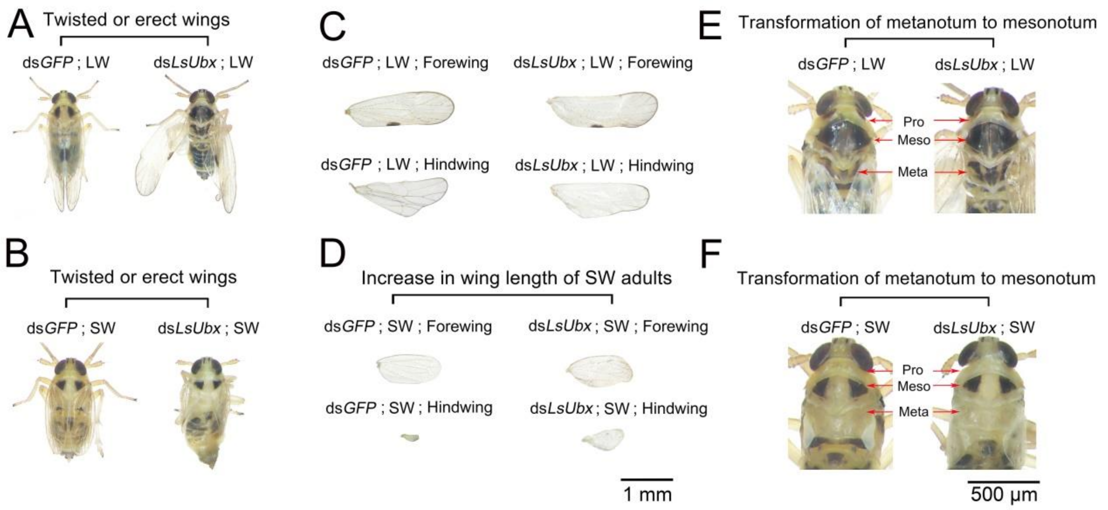

Supplementary Fig. 6 | Phenotypes of *L. striatellus* adults resulting from ds*LsUbx* injection at 3rd instar. (*A*) Twisted or erect wings in the long-wing form (LW). (B) Twisted or erect wings in the short-wing form (SW). (C) Forewing and hindwing of *L. striatellus* LW adults treated with ds*LsUbx*. (D) Forewing and hindwing of *L. striatellus* SW adults treated with ds*LsUbx*. (E) Transformation of metanotum to mesonontum in the LW. (F) Transformation of metanotum to mesonotum in the SW. Pro, Pronotum; Meso, Mesonotum; Meta, Metanotum.

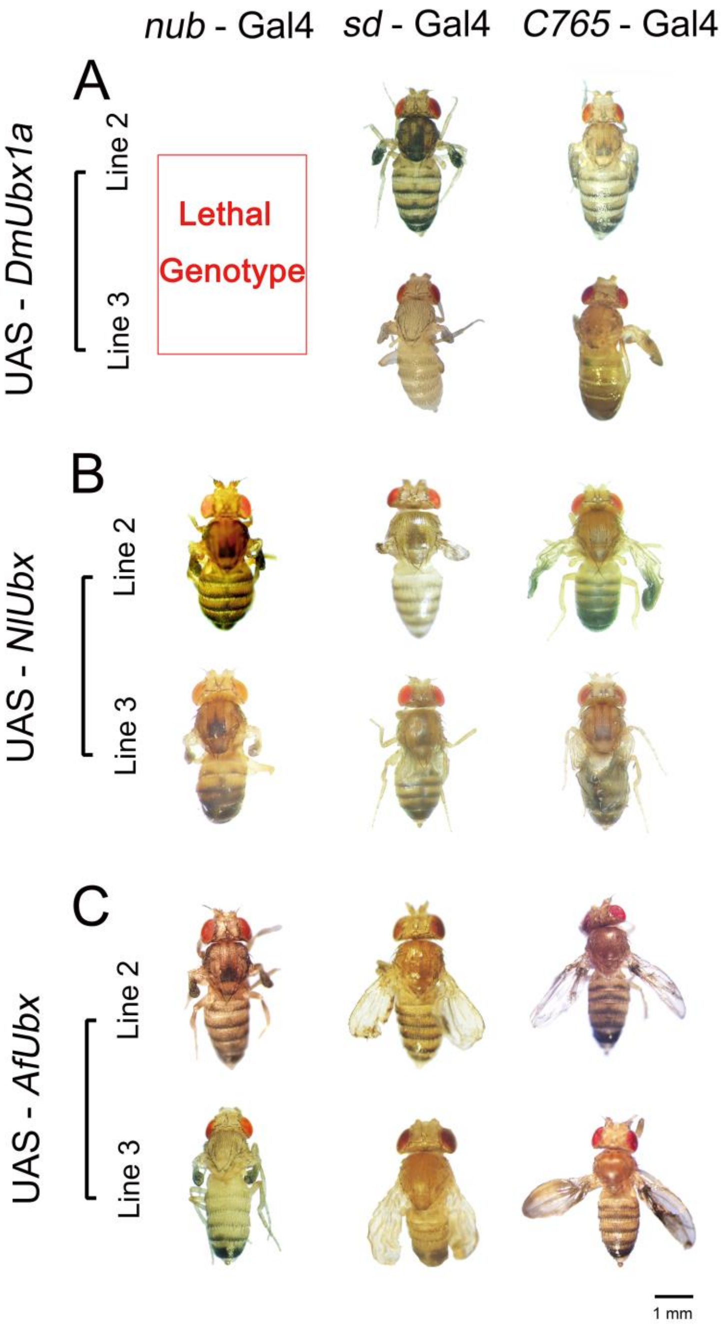

Supplementary Fig. 7 | Additional examples of transgenic fly lines expressing Ubx orthologues in the wing discs driven by *nub*-Gal4 (left), *sd*-Gal4 (middle), or *C765*-Gal4 (right). (A) Adults of different transgenic fly lines expressing *DmUbx1a*. (B) Adults of different transgenic fly lines expressing *NlUbx*. (C) Adults of different transgenic fly lines expressing *AfUbx*. *DmUbx1a*: *Drosophila melanogaster Ubx*; *NlUbx*: *Nilaparvata lugens Ubx*; *AfUbx*: *Artemia franciscana Ubx*.

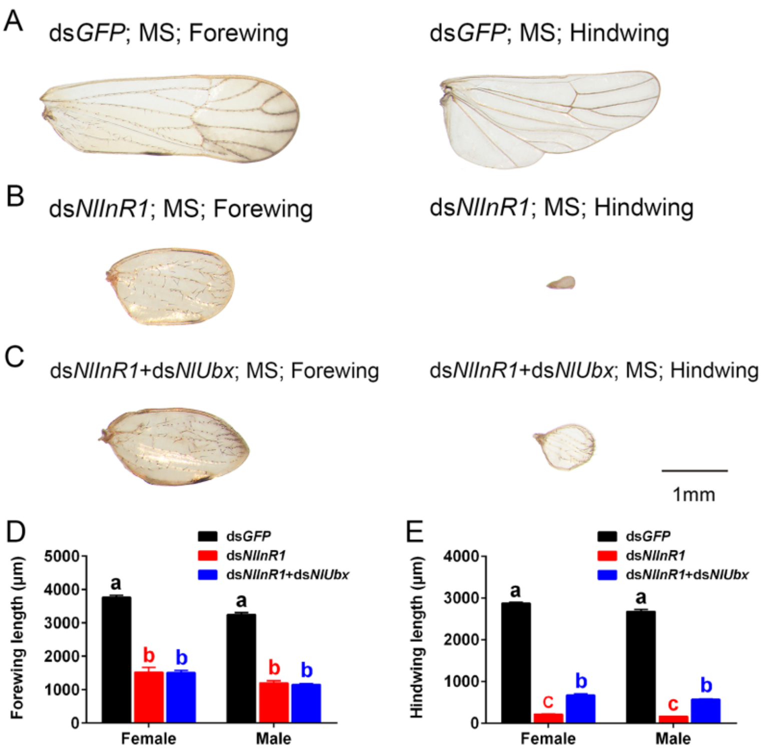

Supplementary Fig. 8 | Phenotypical changes of *N. lugens* adults resulting from *NlInR1* single RNAi and *NlInR1*/*NlUbx* double RNAi. dsRNAs were injected into nymphs from MS at 3rd instar. (A) The forewing and hindwing of adults resulting from nymphs of MS treated with ds*GFP* injection. (B) The forewing and hindwing of adults resulting from nymphs of MS treated with ds*NlInR1* injection. (C) The forewing and hindwing of adults resulting from nymphs of MS treated with ds*NlInR1*/ds*NlUbx* double injection. The forewing length (D) and hindwing length (E) of adults emerged from nymphs of MS treated with ds*NlInR1* or with ds*NlInR1*/ds*NlUbx* double injection. Different letters on the columns in (D) and (E) indicated significant differences among the treatments using ANOVA and LSD analysis ($P < 0.05$). At least 9 survival adults with phenotypic changes were measured. MS: Macropterous Strain; BS: Brochypterous Strain.
